# Naproxen and Diclofenac Attenuate Atorvastatin-induced Preconditioning of the Myocardium

**DOI:** 10.7759/cureus.1201

**Published:** 2017-04-29

**Authors:** Zoltan Varga, Martina Nemcekova, Slavka Carnicka, Veronika Slezakova, Miriam Petrova, Marek Majdan, Tana Ravingerova, Viera Kristova

**Affiliations:** 1 Internal Medicine Residency, Florida Hospital Orlando; 2 Institute for Heart Research, Slovak Academy of Sciences; 3 Department of Pharmacology and Clinical Pharmacology, Comenius University in Bratislava; 4 Department of Public Health, Trnava University

**Keywords:** preconditioning, cyclooxygenase-2, atorvastatin, naproxen, diclofenac, myocardial infarction

## Abstract

Statins reduce infarct size (IS) in ischemia-reperfusion injury of the myocardium. Inhibition of cyclooxygenase-2 (COX-2) attenuates this benefit. We investigated the effect of two widely used non-selective non-steroidal anti-inflammatory drugs (NSAIDs) with different degree of anti-COX-2 activity on atorvastatin-mediated preconditioning. Wistar rats received oral atorvastatin (10 mg∙kg^-1^∙day^-1^), naproxen (10 mg∙kg^-1^∙day^-1^), diclofenac (8 mg∙kg^-1^∙day^-1^), atorvastatin+naproxen, atorvastatin+diclofenac or water for three days. Hearts were then excised and perfused in the Langendorff system. Area at risk (AR) and IS were determined after 30 min of regional ischemia and 120 min of reperfusion. Atorvastatin reduced IS by 51.3% compared with controls (14.7 ± 3.9% vs. 30.2 ± 4.6% of the AR; *P* < 0.001). Naproxen and diclofenac alone did not alter IS compared to control. Diclofenac completely abrogated atorvastatin-mediated protection of the myocardium. Naproxen significantly attenuated but did not eliminate the IS reducing the effect of atorvastatin when compared with controls (*P* = 0.038). The difference in IS between the atorvastatin+naproxen group and the atorvastatin+diclofenac group showed a strong trend in reaching statistical significance (*P* = 0.058), but was not found to be significant. Our results suggest relatively small, but noticeable differences among non-selective NSAIDs in their potential to attenuate statin-mediated preconditioning.

## Introduction

Inhibitors of 3-hydroxy-3-methylglutaryl coenzyme A reductase (statins) are widely used in the treatment of patients at the time or after various forms of acute coronary syndromes (ACS). Guidelines advocate the early initiation of statin therapy in ACS irrespective of cholesterol levels [1-2]. The benefit of statins is likely achieved partially by cholesterol-independent (pleiotropic) effects [3]. One of these pleiotropic effects is an increase in resistance of myocardium to ischemia-reperfusion (IR) injury.

Guidelines for the management of ST-elevation myocardial infarction call for the investigation of new pharmacological strategies to help minimize the consequences of IR injury. One of the possibilities is to take advantage of the protective effect of statins, which according to guidelines, requires further study [2].

Administration of statins before myocardial ischemia, during ischemia or reperfusion, exhibited an infarct size (IS) limiting the effect in animal experiments [4-7]. Clinical trials suggest that pre percutaneous coronary intervention (pre-PCI) administration of atorvastatin might be of substantial benefit [8-15]. Similarly, positive results were reported after pre-treatment with rosuvastatin [8-9,16-18]. If future studies confirm this data, high dose statin pre-treatment before PCI might become standard practice.

The mechanism of statin-mediated protection has not yet been fully understood, but animal experiments have shown that the activity of cyclooxygenase-2 (COX-2) seems to be necessary, while cyclooxygenase-1 seems to be non-essential. Administration of selective COX-2 inhibitors attenuates the IS limiting effect of statins in animal models of IR injury of the myocardium [5,19]. In humans, selective COX-2 inhibition abolishes the protective effect of rosuvastatin on IR-induced endothelial dysfunction in the radial artery [20]. In rats, acetylsalicylic acid, a non-selective COX inhibitor with dose-dependent anti-COX-2 activity, blunted the IS limiting effect of atorvastatin in a dose-dependent manner [6]. This brings up the possibility of significant differences among individual non-selective non-steroidal anti-inflammatory drugs (NSAIDs) with different anti-COX-2 activity in their potential to interact with the IS-limiting effect of statins.

Aims: 1) To determine, if naproxen and diclofenac, two widely used non-selective NSAIDs with different anti-COX-2 activity [21], interfere with atorvastatin-mediated protection of myocardium from IR injury; 2) to determine, if there are significant differences in the level of attenuation of IS-limiting effect of atorvastatin between the two drugs; 3) to determine the effect of the administered medicine on incidence and severity of IR-induced arrhythmias during the protocol.

## Materials and methods

### Animal care

Experiments were conducted on male Wistar rats (Department of Toxicology and Laboratory Animals Breeding Detached Branch, Dobra Voda, Slovakia) (body weight of 300 ± 19 g), which were fed a standard diet, tap water ad libitum and received humane care in accordance with the guide for the care and use of laboratory animals (Eight edition, NRC 2011). The study was approved by the Ethics Committee of the Institute for Heart Research of the Slovak Academy of Sciences and by the Animal Health and Animal Welfare Division of the State Veterinary and Food Administration of the Slovak Republic.

### Materials

Naproxen, diclofenac, thiopental and heparin were purchased from Sigma-Aldrich (Prague, Czech Republic). Atorvastatin was purchased from Zentiva Slovakia (Bratislava, Slovakia).

### Pre-treatment

All drugs were dissolved in water and administered for three days by oral gavage. The three-day duration of pre-treatment was chosen for comparability and consistency since this duration was used in most prior similar experiments. Control animals received water by oral gavage. Rats were randomly divided into six groups: group one (control), group two (atorvastatin), group three (naproxen), group four (diclofenac), group five (atorvastatin+naproxen) and group six (atorvastatin+diclofenac). The drugs were administered once daily in the following doses: atorvastatin – 10 mg∙kg-1∙day-one, naproxen - 10 mg∙kg-1∙day-one, diclofenac - 8 mg∙ kg-1∙day-one. On the fourth day, rats were euthanized and their hearts excised.

The selected doses of drugs are in line with those used in prior experiments in animal models; the doses of naproxen and diclofenac administered provide approximately equipotent analgesic/anti-inflammatory effects according to published data [5-7,22-25].

### Perfusion protocol

Rats were anesthetized by intraperitoneal application of thiopental (60 mg∙kg-1) + heparin (500 IU). After the onset of deep anesthesia, hearts were rapidly excised and placed into cold perfusion buffer. Hearts were perfused in the Langendorff system after cannulation via the aorta at a constant pressure of 70 mm Hg at 37 ºC. Perfusion solution was a modified Krebs-Henseleit buffer gassed with 95% oxygen (O2) and five percent carbon di oxide (CO2) (pH = 7.4) containing (in mmol∙L-1) sodium chloride (NaCl) (118.0), potassium chloride is (KCl) (3.2), magnesium sulfate (MgSO4) (1.2), sodium bicarbonate (NaHCO3) (25.0), monopotassium phosphate (KH2PO4) (1.18), calcium chloride (CaCl2) (2.5) and glucose (7.0). Contaminants were removed from the solution via filtering through a 5 μm porosity filter (Millipore, Billerica, Massachusetts, USA). Electrical activity of the heart was registered by means of two electrodes made of stainless steel attached to the apex of the heart and the aortic cannula. Left ventricular (LV) pressure was measured by means of a nonelastic water-filled balloon inserted into the LV cavity through the left atrium and connected to a pressure transducer (MLP844; ADInstruments, Spechbach, Germany). Target end-diastolic pressure was 5-7 mm Hg. LV systolic pressure (LVSP), LV diastolic pressure (LVDiP), LV developed pressure (LVDP; systolic – diastolic), maximal rates of pressure development and fall, +(dp/dt)max and –(dp/dt) max, heart rate (HR; derived from the electrogram) and coronary flow (CF) were measured and recorded throughout the protocol. All parameters and arrhythmias were analyzed with PowerLab/8SP Chart 5 software (ADInstruments, Spechbach, Germany). The ligature (Mersilk black W 582, Johnson & Johnson, Bratislava, Slovakia) for induction of regional ischemia was placed loosely around the left anterior descending coronary artery (LAD), close to its origin, immediately after cannulation of the heart. A traction type plastic occluder was placed on the suture, without compromising blood flow through the artery.

The isolated hearts underwent 15 minutes of stabilization period. Regional ischemia was induced by constricting the myocardium around LAD with the use of the ligature and plastic occluder. Reduction of CF by approximately 40% was targeted. The release of the ligature with subsequent reperfusion was done after 30 minutes. The success of reperfusion was verified by monitoring the increase of CF. The 30 minutes of ischemia were followed by 120 minutes of reperfusion. The ischemia and reperfusion protocol was consistent with our previous experiments [26].

### Determination of infarct size and area at risk

The IS and size of the area at risk (AR) were delineated by double staining with five percent potassium permanganate and 2, 3, 5-triphenyltetrazolium chloride and determined by a computerized planimetric method, as described earlier [26]. Infarct size was expressed both as a percentage of AR and of the left ventricle.

### Arrhythmia quantification

Electrical activity of the heart was recorded during stabilization, ischemia and the first 10 minutes of reperfusion. The recorded electrogram was analyzed in accordance with the Lambeth Conventions [27]. The focus was on quantification of the number of premature ventricular contractions (PVC), incidence and duration of episodes of ventricular tachycardia (VT), four or more consecutive ectopic beats and incidence and duration of ventricular fibrillation (VF).

### Statistical analysis

Data are presented as arithmetic means ± standard deviation (SD). The statistical differences between groups were studied using two-way analysis of variance (ANOVA). Post hoc comparisons were done with Tukey correction for multiple comparisons. The analysis was performed with the use of statistical software SPSS for Windows, version 19 (IBM SPSS Inc. Chicago, IL, USA). As a threshold for statistical significance, a P value of ˂ 0.05 was set.

## Results

The protocol included 57 Wistar rats, each of the groups contained nine or 10 animals. A total of six isolated hearts were excluded from the experiment, three because of no signs of sufficient ischemia after coronary artery ligation, one because of no sign of reperfusion after release of the ligature, one because of permanent total loss of contractile function during the protocol and the last one because of a problem with determination of the size of infarction.

There were no significant differences between the groups in the number of excluded isolated hearts, body weight or in the values of HR, LVSP, LVDiP, LVDP and CF at baseline, i.e., prior to ischemia (Table 1).

**Table 1:** Values (mean ± standard deviation) of heart rate (HR), left ventricular systolic pressure (LVSP), left ventricular diastolic pressure (LVDiP), left ventricular developed pressure (LVDP) and coronary flow (CF) at baseline n = number of animals in the group; ATV - atorvastatin, NAP - naproxen, DIC - diclofenac

	Control	NAP	DIC	ATV	ATV+NAP	ATV+DIC
*N*	9	8	8	8	9	9
HR (beats/min)	283 ± 28	255 ± 38	270 ± 27	266 ± 47	281 ± 60	280 ± 40
LVSP (mmHg)	97.2 ± 16.6	89.1 ± 10.5	87.8 ± 13.1	87.9 ± 10.5	86.5 ± 10.1	80.0 ± 16
LVDiP (mmHg)	5.1 ± 2.2	7.1 ± 4.7	6.1 ± 0.7	4.4 ± 1.7	6.0 ± 1.4	6.4 ± 1.4
LVDP (mmHg)	92.0 ± 18.5	82.0 ± 13.3	81.7 ± 13.3	83.5 ± 11.2	80.5 ± 11.2	73.6 ± 16.7
CF (ml/min)	9.5 ± 1.2	8.0 ± 2.3	8.4 ± 1.3	7.3 ± 1.1	7.3 ± 1.2	8.8 ± 2.5

### Infarct size and area at risk protocol

No significant differences in body weight and AR were found between the groups (Table 2). 

**Table 2:** Body weight, volume of area at risk (AR), size of area at risk shown as percentage of the left ventricle (LV), infarct size (IS) shown as percentage of the area at risk and infarct size shown as percentage of the left ventricle Data shown as mean ± standard deviation; n = number of animals in the group; ATV - atorvastatin, NAP - naproxen, DIC – diclofenac

	Control	NAP	DIC	ATV	ATV + NAP	ATV + DIC	*P *value
*N*	9	8	8	8	9	9	
Body weight, g	300 ± 19	301 ± 18	299 ± 11	303 ± 22	297 ± 19	302 ± 23	0.985
AR volume, mg	440 ± 65	521 ± 83	432 ± 67	414 ± 104	412 ± 86	456 ± 110	0.352
AR (% of LV)	49.7 ± 8.2	53.1 ± 8.9	52.9 ± 9.3	50.9 ± 7.9	52.7 ± 9.8	52.4 ± 11.3	0.967
IS (% of AR)	30.2 ± 4.6	30.5 ± 2.6	29.9 ± 4.4	14.7 ± 3.9	24.0 ± 5.3	29.8 ± 3.9	<0.001
IS (% of LV)	15.0 ± 3.6	16.2 ± 3.1	16.0 ± 4.7	7.2 ± 1.2	12.5 ± 2.7	16.6 ± 4.6	<0.001

**Figure 1:**
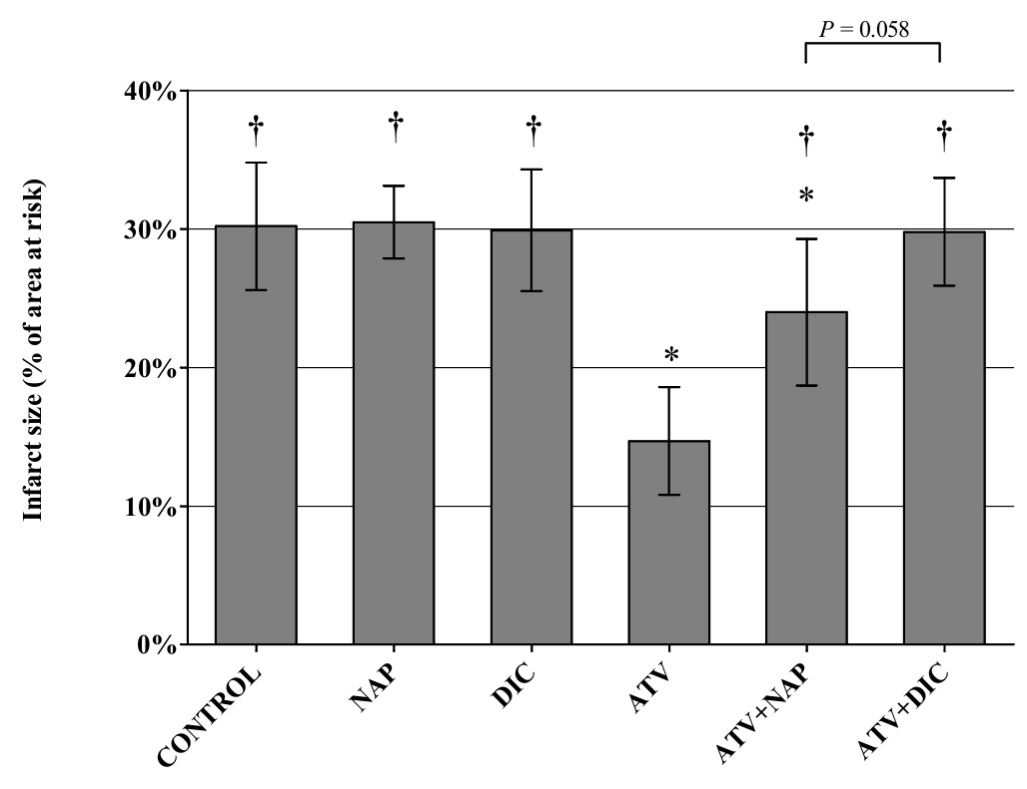
Infarct size, calculated as percentage from the area at risk after 30 minutes of ischemia and 120 minutes of reperfusion Values shown as mean ± standard deviation. N = control - 9, naproxen (NAP) - 8, diclofenac (DIC) - 8, atorvastatin (ATV) - 8, ATV+NAP - 9, ATV+DIC - 9; † - P < 0.05 when compared to ATV, * - P < 0.05 when compared to controls

Infarct size calculated as percentage of the area at risk, did not differ from control (30.2 ± 4.6%) for naproxen (30.5 ± 2.6%) or diclofenac (29.9 ± 4.4%) (P = 0.99 for both) (Figure 1).

Pre-treatment with atorvastatin (14.7 ± 3.9%) significantly (P < 0.001) reduced infarct size compared with the control group (Figure 1). The IS was reduced by 51.3%. Both naproxen plus atorvastatin and diclofenac plus atorvastatin displayed significant attenuation of the infarct size limiting effect of atorvastatin alone (P < 0.001 for both) (Figure 1). Atorvastatin mediated reduction of IS was less affected by co-administration of naproxen than co-administration of diclofenac (24.0 ± 5.3% vs. 29.8 ± 3.9%), but the difference did not quite reach statistical significance (P = 0.058). IS in the atorvastatin+diclofenac group was comparable to controls (P = 0.99), but a significantly smaller IS was found in the atorvastatin+naproxen group than in controls (P = 0.038).

**Figure 2:**
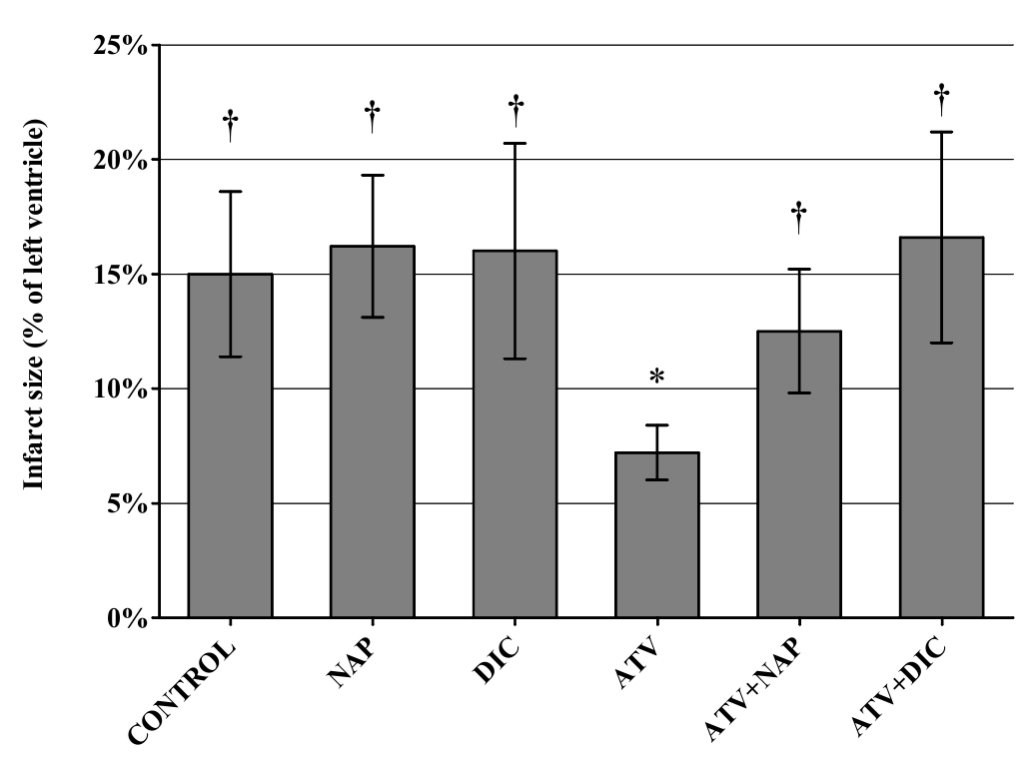
Infarct size calculated as percentage from the volume of left ventricle after 30 minutes of ischemia and 120 minutes of reperfusion Values shown as mean ± standard deviation. N = control - 9, naproxen (NAP) - 8, diclofenac (DIC) - 8, atorvastatin (ATV) - 8, ATV+NAP - 9, ATV+DIC - 9; † - P < 0.05 when compared to ATV, * - P < 0.05 when compared to controls

Analysis of IS calculated as a percentage of left ventricular mass yielded similar results, but there was no significant difference between atorvastatin+naproxen and controls (Figure 2).

### Arrhythmias

Administration of naproxen or diclofenac did not affect the number of PVCs. The overall number of PVCs during the whole protocol was lower in the atorvastatin group than in controls, but without statistical significance (P = 0.334) (Table 3).

**Table 3:** Number of premature ventricular contractions (PVCs), number and duration of episodes of ventricular tachycardia (VT) during the 30 minutes of ischemia (isch) and the first 10 minutes of reperfusion (rep) Data shown as mean ± standard deviation; n = number of animals in the group; ATV - atorvastatin, NAP - naproxen, DIC – diclofenac

	Control		NAP		DIC		ATV		ATV + NAP		ATV + DIC	
*N*	9		8		8		8		9		9	
	isch.	rep.	isch.	rep.	isch.	rep.	isch.	rep.	isch.	rep.	isch.	rep.
Number of PVCs	495 ± 153	296 ± 51	516 ± 259	321 ± 85	456 ± 179	295 ± 87	330 ± 193	252 ± 58	348 ± 188	300 ± 106	372 ± 207	318 ± 117
Number of VT episodes	30.1 ± 17.3	8.7 ± 3,3	30.6 ± 19.9	8.4 ± 2.9	29.6 ± 20.7	8.6 ± 3.7	20.1 ± 14.7	6.9 ± 2.4	19.7 ± 12.3	8.7 ± 3.7	22.2 ± 14.1	8.3 ± 6.1
Duration of VT (s)	39.6 ± 19.9	34.1 ± 16.6	44.1 ± 31.1	39.1 ± 17.9	37.8 ± 21.5	33.6 ± 19.2	24.3 ± 16.9	27.9 ± 11.3	25.6 ± 17.0	36.2 ± 18.7	27.6 ± 18.1	36.9 ± 24.8

The mean number of PVCs in groups atorvastatin+naproxen and atorvastatin+diclofenac was lower than in controls and higher than in the atorvastatin group, but the differences were not statistically significant.

Number of VT episodes during the whole protocol was not influenced by naproxen or diclofenac (Table 3). Although the number of VT episodes in the atorvastatin group was lower than in controls during ischemia, reperfusion and also the total duration of the protocol, the difference was not found significant. A number of VT episodes was similar in the atorvastatin, atorvastatin+naproxen and atorvastatin+diclofenac groups.

Duration of VT episodes was not affected by administration of naproxen or diclofenac (Table 3). No significant reduction of VT episode duration was observed in the atorvastatin group, although duration was shorter than in controls. Duration of VT episodes was similar in the atorvastatin, atorvastatin+naproxen and atorvastatin+diclofenac groups.

An episode of VF occurred in just two isolated hearts, one in the control, and the other in the naproxen group.

## Discussion

In the present study, we demonstrated that three-day pre-treatment with atorvastatin reduced IS by 51.3%. Two non-selective NSAIDs, naproxen and diclofenac, interfered with atorvastatin-mediated cardioprotection. The IS limiting effect of atorvastatin was completely abrogated by diclofenac. Although naproxen significantly attenuated the IS limiting effect of atorvastatin, co-administration of atorvastatin and naproxen still resulted in significantly reduced size of area of myocardial necrosis when compared to controls. The difference in IS between the atorvastatin+naproxen group and the atorvastatin+diclofenac group was close to reaching statistical significance (P = 0.058).

The seemingly greater potential of diclofenac compared to naproxen to alleviate the IS reducing effect of atorvastatin might be related to its more pronounced COX-2 inhibitory activity. Previously, it was shown that a high therapeutic dose of diclofenac suppresses the activity of COX-2 to higher degree than a high therapeutic dose of naproxen [21]. It is known that administration of selective COX-2 inhibitors attenuates statin-mediated, increase in resistance of myocardium to IR [5,19]. Administration of various doses of acetylsalicylic acid, a non-selective inhibitor of COX, dose-dependently blunts the IS limiting effect of atorvastatin [6]. These findings show the importance of the degree of COX-2 inhibition on the interaction between statins and COX inhibitors. Since therapeutic doses of various non-selective NSAIDs block COX-2 to a different extent, significant differences may exist in the potential to decrease statin-mediated cardioprotection in the setting of IR. To our knowledge, no prior study evaluated this hypothesis.

Experiments about effects of statins on IR-induced arrhythmias yielded inconsistent results. Short-term administration of pravastatin elicited protective effect against IR-induced arrhythmias in ex-vivo animal models of IR injury [28]. On the other hand, a single dose of pravastatin two hours before ischemia had no protective effect under in-vivo conditions [29]. In the present experiment, atorvastatin did not offer statistically significant protection from IR-induced arrhythmias. However, the overall number of PVCs and VT episodes was lower and the duration of VT episodes shorter in the atorvastatin group than in controls. The lack of statistically significant antiarrhythmic effect in our experiment is in contrast with previously published results of ex-vivo experiments with other statins. We can speculate that the different outcome might be caused by molecular properties of atorvastatin, by differences in the experimental protocol, or by the relatively small size of the experimental groups leading to potential beta error (β-error). Further research is needed to clarify the antiarrhythmic properties of atorvastatin in the setting of myocardial ischemia.

The results of our experiment could have significant clinical implications. Patients with ischemic heart disease often possess comorbidities that result in short- or long-term treatment with analgesics. NSAIDs are used weekly by 70% of patients over age 65 and 34% of them take a NSAID at least once per day [30]. These data demonstrate the extent that treatment with NSAIDs could have pleiotropic effects of statins. As demonstrated by Liuni, et al., short term rosuvastatin administration protects from IR-induced endothelial dysfunction in humans. Celecoxib, a selective COX-2 inhibitor, blunted this effect [20]. If confirmed in clinical studies, our results bring up the possibility of clinically significant differences between various non-selective NSAIDs in their potential to diminish the benefit of pre-procedural statin administration in the setting of elective or urgent percutaneous coronary intervention (PCI). Naproxen rather than diclofenac might be better suited for analgesic treatment of patients at risk of ACS, because, at least in our animal model, it does not completely abrogate the IS limiting effect of pre-PCI statin administration. According to our results and previously published data, even non-selective NSAIDs with less pronounced anti-COX-2 activity have the potential to, at least partially, attenuate statin-mediated reduction of IS. It might be reasonable to stop administration of such drugs if elective PCI with pre-procedural statin administration is scheduled in a patient.

## Conclusions

In conclusion, we can summarize our findings as follows: 1) Three-day atorvastatin administration led to a significant reduction of IS; 2) Three-day administration of both naproxen and diclofenac significantly attenuated the IS limiting effect of atorvastatin; 3) Diclofenac completely abrogated atorvastatin-mediated protection of the myocardium. In contrast, naproxen, the non-selective NSAID with less anti-COX-2 activity, attenuated the IS reducing effect of atorvastatin, but compared with controls, co-administration of atorvastatin and naproxen still lead to significantly reduced IS; 4) Although, overall numbers of premature ventricular contraction (PVCs) and ventricular tachycardia (VT) episodes were lower in the atorvastatin group, atorvastatin failed to offer significant protection from arrhythmias.

Our results suggest potentially meaningful differences between various non-selective NSAIDs in their potential to attenuate statin-mediated protection from IR injury. Further studies are needed to evaluate the possible clinical significance of these differences in treatment of patients with ischemic heart disease, who are in need of NSAID administration.

The limitations of our study include the ex-vivo design, the fact that COX-2 activity in myocardial tissue after drug administration was not tested, just extrapolated from prior data and the use of only one dosage of each NSAID making impossible to detect dose-dependent changes in effect. Also, we have to mention that direct extrapolation of the results obtained in this animal experiment to human subjects is impossible, although human studies previously discussed in this paper indicate a similar role of COX-2 in ischemia and reperfusion in human and rats.
